# Properties of Mortar with Recycled Aggregates, and Polyacrylonitrile Microfibers Synthesized by Electrospinning

**DOI:** 10.3390/ma12233849

**Published:** 2019-11-22

**Authors:** Manuel J. Chinchillas-Chinchillas, Manuel J. Pellegrini-Cervantes, Andrés Castro-Beltrán, Margarita Rodríguez-Rodríguez, Víctor M. Orozco-Carmona, Héctor J. Peinado-Guevara

**Affiliations:** 1 Faculty of Engineering Mochis, Autonomous University of Sinaloa, Fuente de Poseidón y Ángel Flores s/n, Col. Jiquilpan, Module B2, Los Mochis, Sinaloa 81210, Mexico; andres.castro@uas.edu.mx; 2 Corrosion Department, Advanced Materials Research Center, Chihuahua 31136, Mexico; victor.orozco@cimav.edu.mx; 3 Faculty of Economic and Administrative Sciences, Autonomous University of Sinaloa, San Joachín, Guasave 81101, Sinaloa, Mexico; hpeinado75@hotmail.com

**Keywords:** recycled aggregate, polyacrylonitrile microfibers, electrospinning, durability, carbonation

## Abstract

Currently it is necessary to find alternatives towards a sustainable construction, in order to optimize the management of natural resources. Thus, using recycled fine aggregate (RFA) is a viable recycling option for the production of new cementitious materials. In addition, the use of polymeric microfibers would cause an increase in the properties of these materials. In this work, mortars were studied with 25% of RFA and an addition of polyacrylonitrile PAN microfibers of 0.05% in cement weight. The microfibers were obtained by the electrospinning method, which had an average diameter of 1.024 µm and were separated by means of a homogenizer to be added to the mortar. Cementing materials under study were evaluated for compressive strength, flexural strength, total porosity, effective porosity and capillary absorption, resistance to water penetration, sorptivity and carbonation. The results showed that using 25% of RFA causes decreases mechanical properties and durability, but adding PAN microfibers in 0.05% caused an increase of 2.9% and 30.8% of compressive strength and flexural strength respectively (with respect to the reference sample); a decrease in total porosity of 5.8% and effective porosity of 7.4%; and significant decreases in capillary absorption (approximately 23.3%), resistance to water penetration (25%) and carbonation (14.3% after 28 days of exposure). The results showed that the use of PAN microfibers in recycled mortars allowed it to increase the mechanical properties (because they increase the tensile strength), helped to fill pores or cavities and this causes them to be mortars with greater durability. Therefore, the use of PAN microfibers as a reinforcement in recycled cementitious materials would be a viable option to increase their applications.

## 1. Introduction

Construction industry is characterized by a significant demand of energy and raw materials such as clay, wood, metal, water, petrous materials, etc. [1]. In the construction sector, the aggregate is the predominant material from the volume point of view, substituting the natural fine aggregate (NFA) with recycled fine aggregates (RFA) contributes to the sustainability and conservation of natural resources [2,3,4]. Growing environmental concerns and landfill shortages, overexploitation of rivers and hills ecosystems, increased transportation, and rising landfill costs are the driving forces that promote the recycling of concrete demolition waste to be used in a new substitute material [5,6,7]. Mortar made with recycled aggregates is no longer just a field of research, it is already a practical reality and it has been used for some years in several countries [8]. The amount of construction and demolition waste deposited in landfills differs from one country to another, in Hong Kong, approximately 20 million tons of waste were produced in 2004, only 12% of the waste was disposed of in landfills and 88% was used as filling material. The generation of construction and demolition waste in the European Union (EU) reached 850 million tons in 2008, which represents 31% of total waste generation in the EU [9]. The countries that recycle more construction material in the European Union are Holland, Denmark and Belgium, generating and using waste of 90%, 81% and 87%, respectively [10]. In most research, it is mentioned that the use of a certain percentage of recycled coarse aggregate (RCA) does not damage the durability and the resistance of the cementitious materials, but the use of recycled fine aggregate (RFA) is restricted or even prohibited due to its unsatisfactory properties [11,12]. However, several studies suggest that its use is not very unfavorable and that results similar to the ones obtained with natural fine aggregates with low substitutions can be obtained [13]. According to some studies, replacing less than 20%–30% RFA does not cause a significant decrease in the physical and mechanical properties of the mortar [14,15]. Therefore, to contribute to sustainability, it is necessary to reuse these materials and to develop new mortars with less effects on the resistance and durability properties of the structures made with cementitious materials.

Mechanical properties represent the capacity of mortar to support stress [16] and they are related to the durability properties, which are indicators of the deterioration suffered by mortar due to some external effect, and it depends on the ability of a fluid to penetrate the microstructure of mortar allowing the introduction of molecules (carbon dioxide and chloride ions) that react and destroy the chemical stability [11]. In order to improve these properties, diverse researchers use additives (superplasticizers, accelerators, etc.), pozzolans (silica fume, fly ash, granulated slag, etc.), nanoparticles (silica, titanium, calcium carbonate, etc.), pieces of polymeric materials (rubber, PET bottles, polypropylene, etc.) and fibers of different nature (fiberglass, steel fibers, polymer fibers, etc.) [17,18,19,20,21,22,23,24,25,26]. In the last decades, fiber reinforced mortar has been used in applications such as pavements, coatings, repair of hydraulic structures patches, thin sheets, prefabrications, projected mortar, reinforcement of slabs, wall cladding, bridge decks, earthquakes, resistant structures, etc. [27]. Currently, it is well established that fiber incorporation improves the engineering performance of cementitious materials including a better resistance to cracking, an increase on ductility and toughness, as well as an improvement on fatigue and impact resistance [28]. Steel fibers are very expensive and produce corrosion [29], glass fibers are very fragile and do not transmit elasticity to the mortar, [30], therefore, polymer fibers are the best option, they are produced at a low cost, they have high elastic properties and they are easy to place in the mortar mixture, improving its dispersion [31], in addition to being the least studied in the construction engineering area. Some of the fibers used as reinforcement of cementitious materials are polypropylene fibers [32], polyethylene terephthalate (PET) fibers [33], polyvinyl alcohol (PVA) fibers [34], among others, but few studies have been done with polyacrylonitrile (PAN) reinforcing fibers [35]. PAN is a polymer widely used in the industry, and has been widely used for the synthesis of microfibers because it has good mechanical properties, easy spinning, chemical stability and high durability [36]. A very efficient method to synthesize PAN microfibers is electrospinning, it is a simple procedure and microfibers are obtained without defects and with diameters ranging from nanometer to micrometer [37,38]. This research studies the effect of the addition of PAN fibers at 0.05% (in cement weight) in the mechanical properties and in the durability in mortars made with 25% of recycled fine aggregates (replacing the natural aggregate), evaluating the resistance to compression, to bending and to water penetration, total porosity, effective porosity, resistance to, capillary absorption, sorptivity and carbonation, since the mechanical properties and durability are the most important measures to be taken into account to observe the useful life of mortar for future applications.

## 2. Materials and Methods

### 2.1. Materials

**Figure 1:**
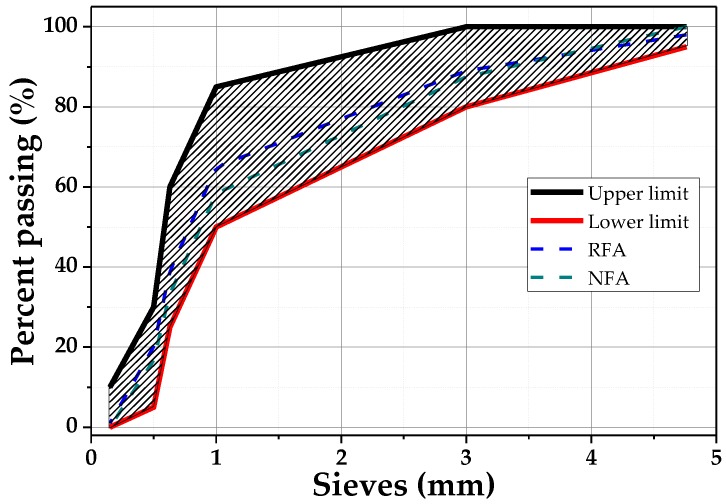
Granulometry of NFA and RFA following the ASTM C-33 standard.

For the preparation of mortars, Portland Cement type III (30R) Cemex^®^ brand (Monterrey, México) was used, which achieves a compressive strength of 30 MPa at 28 days (NMX-C-061) [39], distilled water, NFA and RFA was used, which was obtained by crushing waste concrete by means of a ball mill. To obtain PAN microfibers, PAN (Sigma-Aldrich, Estado de México, México) with a molecular weight of 150,000 g/mol^−1^ and dimethylformamide (DMF) of 99.85% purity was used. Figure 1 shows the granulometric curves of the aggregates, following ASTM C-33 [40]. In Table 1, the properties of the materials used in the manufacture of the mortars are shown.

### 2.2. Production of Recycled Fine Aggregate

The concrete of origin used for the production of the RFA was obtained from a quality control laboratory of a concrete company, which had been assigned to a road paving project with an average resistance of 23 MPa, the test cylinders were stored and free from contaminants. It was necessary to screen the material by means of mechanical crushing using a jaw crusher Maneklal Global Exports model JS-0804 (Los Mochis, México) to minimize the size of the samples. This process was carried out several times until an approximate size of 3 cm was obtained. The last step was to place the pieces of crushed concrete in a ball mill with a Falcon engine model 7518081PA56C (Culiacán, México) with 8–12 mm steel balls for 30 min until obtaining fine material with a maximum size of 0.6 cm, this fine aggregate was subjected to two washing times to eliminate possible contaminating materials. All the material was sieved to comply with the granulometry following the ASTM C-33 standard [40].

### 2.3. Production of PAN Microfiber by Electrospinning

The details of the electrospinning equipment can be observed in the Chinchillas et al. 2019 investigation. The electrospinning parameters were: 17 cm separation between the tip of the needle and the collector plate, 16 kV applied voltage and 1 mL/h flow. The polymeric solution of PAN, was elaborated by adding 12% in PAN weight and 88% of DMF in a vial and stirring for 12 h in room temperature, until a completely homogeneous solution was achieved. This solution was put into a 3 mL syringe and installed in the infusion pump of equipment, the process was started and the fibers were deposited in an aluminum container. 

#### Dispersion of Microfibers in Aqueous Medium

To separate the PAN microfibers, a Branson ultrasonic tip model 450A (Chihuahua, México) was used for the homogenization process. First, the water from each mixture was put into a 1000 mL vial. Then 0.05% of the fibers were added and the ultrasound tip was placed into the vial for 3 hours with an amplitude of 45% to 50% until dispersion was achieved. 

### 2.4. Design of Mortar Mix

In Table 2, the four mortar mixtures are described, showing the amount of materials needed to make the specimens, each mixture corresponds to a mortar volume of 0.000256 m^3^. A water/cement ratio (w/c) of 0.58 was used, after verifying that it was the ideal ratio to obtain a mortar with a plastic consistency, the method used is described in ASTM C1437 [41] (by this procedure, it is possible to obtain a percentage of the workability of the mixture and know if it has a dry, plastic or wet workability [42]). The aggregate/cement ratio (ag/c) used was 2.75, following ASTM C109 [43]. It is worth mentioning that the RFA was previously saturated with water to avoid the absorption of mixing water [44].

The nomenclature used in the mixtures is classified as follows: the first three letters correspond to the origin of the fine aggregate (RFA and NFA), in the case of the RFA it refers to the mixture with a 25% replacement and the mixture with NFA refers to the 100% of natural aggregate used. The samples with the acronym PAN, refer to the addition of microfibers of PAN in 0.05% with respect to the weight of the cement.

### 2.5. Preparation of Specimens and Test Procedures

The morphology and average size of PAN microfibers were observed by scanning electron microscope (SEM), FEI Nova Nano-SEM brand (Chihuahua, México) at 5 kV and at a working distance of 10 mm. The vibration of the polymer bonds was analyzed by infrared spectroscopy (FT-IR) Bruker Alpha II brand (Los Mochis, México) in a range of 4000–500 cm^−1^. The thermogravimetric analysis and differential calorimetry scanning (TGA/DSC) was carried out by using SDT Q600 TA INSTRUMENTS (Los Mochis, México), from 0 to 700 °C with a heating rate of 10 °C/min in nitrogen environment.

Table 3 shows the summary of the tests, the shapes and sizes of the specimens, the curing time, and the norms followed in this research. In all the tests, three specimens were made for each group of samples, and the mixing procedure was following the ASTM C192 standard [45]. For durability tests, it was necessary to cut the cylinders using a Schneider cutter model 55-CO210 (Los Mochis, México) and in accelerated carbonation test, the penetration of CO_2_ was measured through a 1% phenolphthalein solution in water and alcohol and the parameters of the carbonation chamber were 4% of CO_2_, 56% relative humidity and a temperature of 25 °C. 

## 3. Results and Discussion

### 3.1. Microfibers of PAN by Electrospinning

**Figure 2:**
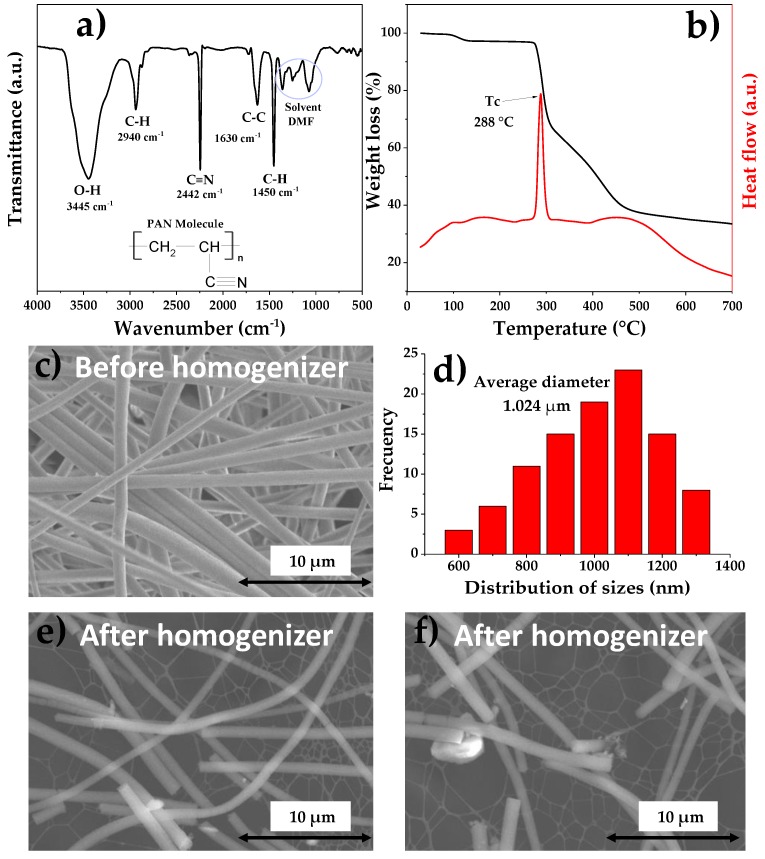
Characterization and separation of PAN microfibers: (**a**) FT-IR, (**b**) TGA/differential calorimetry scanning (DSC), (**c**) SEM, (**d**) average diameter and (**e**,**f**) microfibers after separation.

Figure 2 shows the characterizations that were made to the PAN microfibers obtained by electrospinning and the results after separation by means of the homogenization process. The FT-IR study was carried out in order to know the purity of PAN microfibers, when analyzing the vibration of the polymer links. With this study it is possible to determine if there is a certain amount of the solvent used in the manufacture of microfibers, or if there is any vibration of contaminating molecules. At 3445 and 2940 cm^−1^, the vibrations of the bonds O–H and C–H respectively are observed, due to water absorption [51]. The stretching vibrations of C≡N, C–C and C–H representative of the PAN were observed at 2442, 1630 and 1450 cm^−1^ [52,53]. These results confirm that the structure of the microfibers is PAN. You could also observe some bands at approximately 1368, 1256 and 1088 cm^−1^, which are characteristic of DMF, a solvent used in the synthesis of PAN microfibers [54,55]. On the other hand, the thermogravimetric analysis is shown in Figure 2b. PAN microfibers showed a total weight loss of 87.3% at 700 °C. The chemical decomposition of the polymer begins at approximately 250 °C, and an exothermic peak is observed at 288 °C in the DSC analysis [56]. This confirms that changes in molecular structure occur due to degradation of the material [57]. On the other hand, through SEM images, it is possible to observe the morphology of the microfibers and using “ImageJ” software (version number: 1.51, Wayne Rasband, National Institutes of Health, Bethesda, MD, USA), it is possible to calculate the average diameter. In Figure 2c, PAN microfibers are continuous, and smooth and defect-free morphology is shown, confirming that the parameters used for its fabrication were adequate. The diameters obtained were from 600 nm to 1.3 µm, presenting an average diameter of 1.024 µm (Figure 2d). Recent research has reported PAN microfibers with 1 µm diameter, using the same parameters [58]. Microfibers obtained by electrospinning are separated from each other, but continuous, i.e., it is not possible to control the length after the electrospinning process. So, it is not possible to incorporate them into the cement mortar, which is why a separation and cutting process through a homogenization process is needed. In Figure 2e,f, the cut and separated microfibers can be observed where no change in the microfiber shape is watched, but they are no longer continuous microfibers, now they are shorter (approximate sizes of 15–30 µm), comparable to some studies reported in literature, where microfibers within micrometer scale length are used in cementitious materials [59]. These results confirm that the homogenization process is useful for the separation and cutting of electro-spun microfibers.

### 3.2. Mortar

#### 3.2.1. Compressive and Flexural Strength

**Figure 3:**
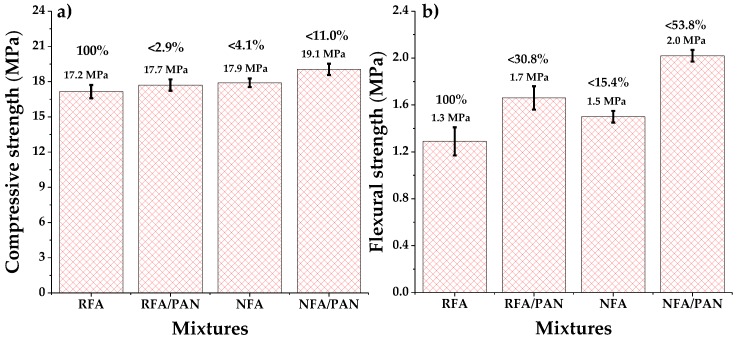
Mechanical properties of mortars: (**a**) compressive strength and (**b**) flexural strength.

The use of recycled aggregates for the manufacture of mortars causes a decrease in their mechanical properties, generating materials with low density and high porosity [60]. Figure 3a shows the results of compressive strength. It can be seen that the mixture with RFA reached a compressive strength of 17.2 MPa, it is the sample with lower mechanical properties of the study, this is attributed to the use of recycled materials (high porosity) [7]. On the other hand, the incorporation of PAN microfibers in recycled mortars caused a slight increase of 2.9%, since microfibers help to withstand internal stresses in cementitious materials [61]. In addition, the NFA mortars reached a value of 17.9 MPa, a result similar to other mortar works with the same ratio w/c = 0.58) [62]. By incorporating PAN microfibers into natural mortars, it increased by 11% compared to the RFA sample. Similar results are observed in Figure 3b (flexural strength), where the RFA mixture was the one that reached a lower value (1.3 MPa). As well as using 0.05% of PAN microfibers in recycled and natural mortars caused an increase of 30.8% and 53.8% respectively. It should be noted that the flexural strength results are more outstanding than the compressive strength, because microfibers have a greater effect on tensile stresses, which are more abundant in flexion [63]. These results confirm that the use of recycled aggregates in the manufacture of mortars causes decreases in mechanical properties due to their high porosity and confirms that the addition of PAN microfibers in small percentages (0.05%) causes significant increases in the mechanical properties of mortars, due to the ability of microfibers to withstand stresses, retain crack propagation and decrease their porosity [64].

#### 3.2.2. Total and Effective Porosity

**Figure 4:**
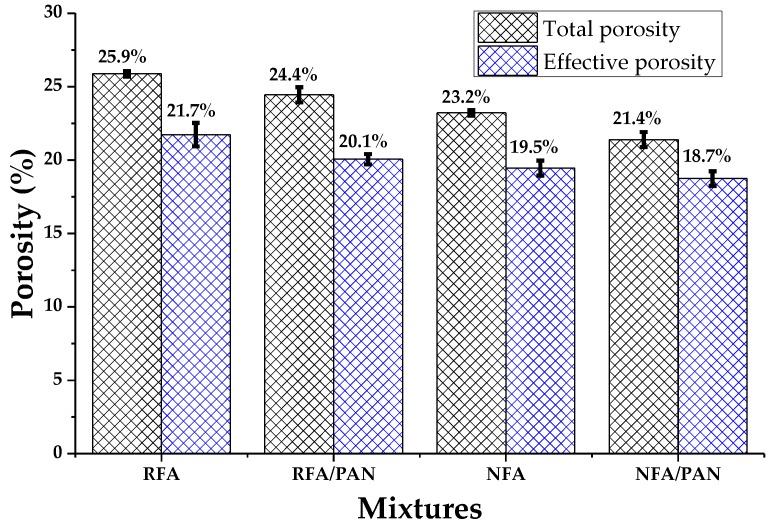
Test of total porosity and effective porosity of the mortar.

Total porosity refers to all the pores found throughout the mortar structure and the effective porosity to the interconnected pores in the cement matrix [65]. Figure 4 shows the result of the total porosity and the effective porosity of the mortars evaluated in this investigation. As expected, the RFA mixtures were the ones that reached the highest values, 25.9% and 21.7% of total porosity and effective porosity respectively. This is due to the fact that the recycled aggregate contains old high-porosity paste in its structure [66]. It was also observed that adding PAN microfibers in the mortar with recycled aggregate (RFA/PAN) caused a decrease in its porosities of 5.8% and 7.4% for total and effective porosity respectively (referring to the RFA mortar). On the other hand, the NFA mixture had similar values to other investigations for mortars with this ratio w/c = 0.58 [48,67]. Finally, the NFA/PAN mortars had a total porosity of 21.4% and an effective porosity of 18.7%, comparing this sample with RFA, the addition of microfibers caused a decrease of 17.4% and 13.8% respectively. As it is known, the use of recycled aggregates causes the cementitious materials to increase their porosities (due to the nature of the aggregate), but the outstanding thing is that with the use of PAN microfibers the mortars decreased their porosities, this is attributed to the ability of the fibers to fill cavities or function as a barrier, preventing the penetration of water into the structure of the material [31].

#### 3.2.3. Capillary Absorption and Resistance to Water Penetration

**Figure 5:**
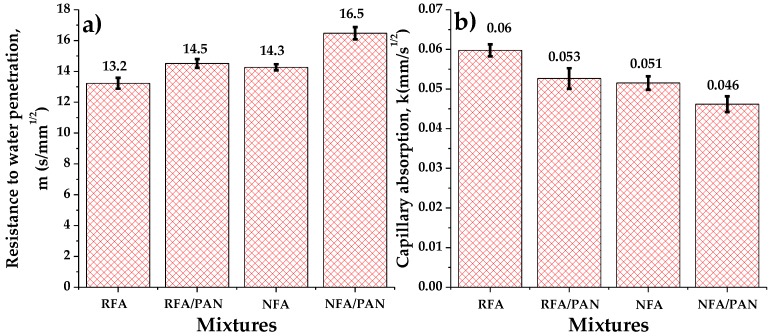
(**a**) Resistance to water penetration and (**b**) capillary absorption of the mortar mixtures.

The absorption and resistance to water penetration are important indicators of the durability of hardened mortar. Reducing water absorption and increasing its resistance to water penetration greatly improve the long-term performance and the useful life of mortar in aggressive environments [68]. Figure 5a shows the results of the resistance to water penetration, and Figure 5b the capillary absorption of the mixtures. It is observed that the RFA mortar had a lower resistance to water penetration and a greater capillary absorption compared to other samples. This is because this type of aggregate contains old mortar adhered to its structure, being a highly porous and very absorbent material [69]. However, it is also observed that adding PAN microfibers to the RFA and NFA caused an increase in resistance to water penetration and a decrease in capillary absorption. This is because microfibers serve as a filler material or as a barrier between the cementitious matrix and the aggregates present in mortar [70]. These results show that the addition of PAN microfibers helped to provide a denser cementitious matrix and consequently increased its durability.

#### 3.2.4. Sorptivity

**Figure 6:**
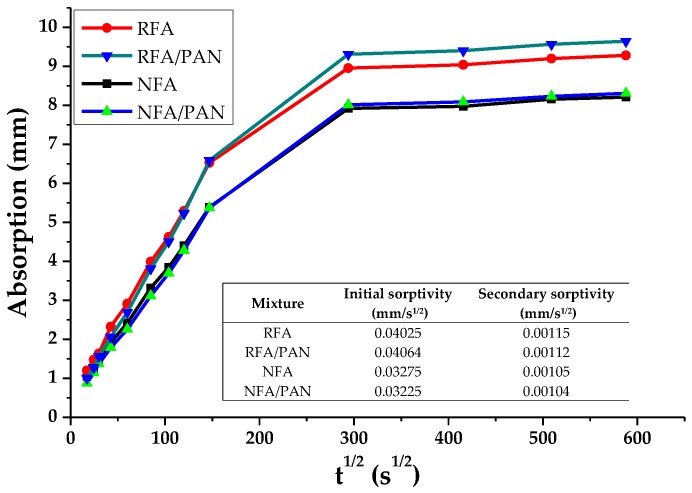
Sorptivity.

Sorptivity is a property of the materials that measures the tendency of a porous material to absorb and transmit water through capillarity [71,72]. Figure 6 shows the results of the absorption capacity of the mortars studied, highlighting that the samples with recycled aggregates were those that had higher absorption values due to the high water retention capacity of the recycled aggregate and its porous structure [73]. It can also be seen that the use of PAN microfibers causes a decrease in the specificity of sorptivity of cementitious materials. This result, in addition to those shown above, demonstrates that PAN microfibers were effectively preventing the flow of water in the mortar microstructure helping to reduce its porosity.

#### 3.2.5. Carbonation

Carbonation is one of the most important studies of durability in cementitious materials and is caused by the reaction that occurs in CO_2_ and moisture inside the pores of cementitious materials [74]. 

**Figure 7:**
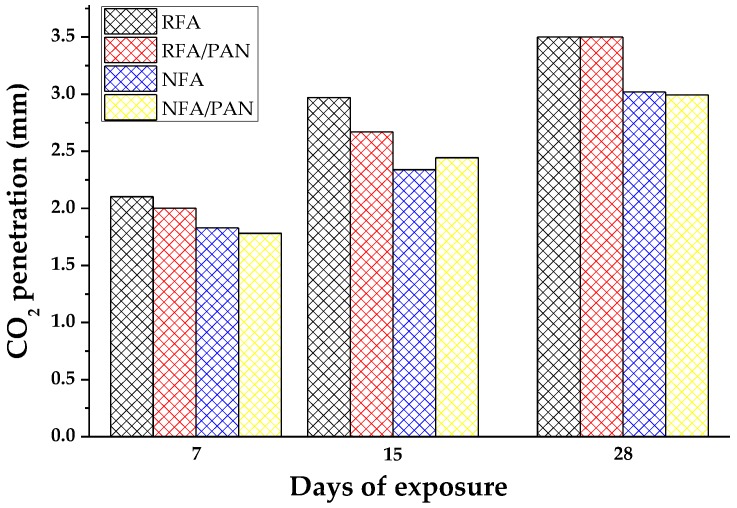
Carbonation results at different days of exposure (7, 15 and 28 days).

The results of CO_2_ penetration in mortars made with RFA and PAN microfibers are shown in Figure 7. In which a clear trend was observed and it matched with the results shown above. At 7 days of exposure, the RFA mixture was the one with the highest CO_2_ penetration (2.1 mm), due to its porous nature, on the other hand, the RFA/PAN mixture slightly decreased the penetration of CO_2_ to 2 mm, because PAN microfibers cause a lower porosity reflecting a lower carbonation. On the other hand, the NFA mixture had a CO_2_ penetration of 1.82 mm and by adding PAN microfibers helped reduce carbonation to 1.75 mm. The same trend was observable in the results at 15 and 28 days of exposure, demonstrating that PAN microfibers helped to counteract the decreases that were generated with the use of RFA in mortars [75].

## 4. Conclusions

The formation of cementitious materials with the use of recycled aggregates opens up a world of possibilities and applications towards sustainable construction. The use of RFA in cementitious materials caused a decrease in the mechanical and durability properties of mortars, due to the large amount of old adhered paste, its high porosity, its high absorption and because it is less resistant than NFAs. However, this research showed an alternative to counteract this problem. The use of PAN microfibers caused an increase in the mechanical properties of compression and flexural strength of 2.9% and 30.8% respectively, it also caused a porosity decrease by 5.8% and 7.4% with respect to the total and effective porosity. By decreasing the porosity of the mortars with the use of PAN microfibers, the capillary absorption decreased by 23.3% and the resistance to water penetration increased by 25%. Finally, carbonation decreased 14.3%. It should be noted that PAN microfibers have not been reported in the literature as a reinforcement of recycled mortars. These results were due to the fact that PAN microfibers provide mortar with tensile strength, help fill cavities or pores within the mortar microstructure and prevent the spread of cracks. Therefore, the use of PAN microfibers as a reinforcement for recycled mortars opens up a world of possibilities for use in future constructions and could be an alternative for sustainable construction.

## Figures and Tables

**Table 1:** Properties of the materials used.

Properties	NFA	RFA	PAN Microfibers	Cement
Diameter range (µm)	-	-	0.6–1.3	-
Average microfiber diameter (µm)	-	-	1.024	
Modulus of elasticity (GPa)	-	-	2.5	-
Hardness (GPa)	-	-	0.48	-
PAN Molecular weight (g/mol)	-	-	150,000	-
PAN concentration (% by weight of DMF)	-	-	12	-
Specific gravity (kg/m^3^)	2.56	2.15	-	3.150
Moisture (%)	6.15	3.04	-	-
Water absorption (%)	4.16	13.63	-	-
Fineness modulus	2.88	3.04	-	-
Type	Natural	Recycled	Polymeric	30R

**Table 2:** Mixture design.

Mixtures	% of RFA	% of NFA	% of Microfibers	Water (l)	Cement (kg)	NFA (kg)	RFA (kg)	PAN Microfibers (kg)	Mix Flow (%)
RFA	25	75	0	2.847	4.908	10.124	3.374	-	116.6
RFA/PAN	25	75	0.05	2.847	4.908	10.124	3.374	0.00245	115.7
NFA	-	100	0	2.847	4.908	13.499	-	-	117.2
NFA/PAN	-	100	0.05	2.847	4.908	13.499	-	0.00245	115.5

**Table 3:** Tests, shape and size of the specimens, curing time and standards applied.

Test	Form of the Specimen (type)	Dimensions of the Specimen (cm)	Curing Time (days)	Standard/Reference
Flexural strength	Prisms	4 × 4 × 16	28	ASTM C 348 [46]
Compressive strength	Post-test pieces of mortar bending	4 × 4	28	ASTM C 349 [47]
Total porosity	Cubes	5 × 5	28	[48]
Effective porosity	Cylinders	7.5 in diameter and 8 in height	28	ASTM C 1585 [49]
Resistance to water penetration and capillary absorption	Cubes	4 × 4	-	[35]
Accelerated carbonation	Cylinders	7.5 in diameter and 2 in height	7, 15 and 28	[50]
